# Influence of Mixing Water Content and Curing Time on Bond Strength of Clinker Masonry: The Wrench Test Method

**DOI:** 10.3390/ma16062171

**Published:** 2023-03-08

**Authors:** Jan Kubica, Iwona Galman

**Affiliations:** Department of Structural Engineering, Silesian University of Technology, 44-100 Gliwice, Poland

**Keywords:** mortar mixing water content, curing time, bond strength, clinker units, IRA, wrench test

## Abstract

In the present study, experimental investigations on the influence of mixing water content used for the preparation of mortar mix using factory-made dry-mix mortar dedicated to bricklaying with clinker masonry units are presented, as well as the curing time on flexural bond strength of masonry made of these two materials. The flexural bond strength was tested using the “wrench test” method. The masonry tests specimens were prepared using three volumes of mixing water as follows: 4.0 L (the value recommended by the mortar manufacturer); 4.5 L; and 5 L of tap water per one 25 kg bag of dry pre-mixed mortar. The influence of the mixing water content was analyzed in relation to curing time. All masonry specimens were tested in four series after 9, 14, 21, and 28 days of sample curing. The results showed that the use of 6 and 18% more mixing water than recommended by the manufacturer (4.5 and 5 L per bag) adversely affected flexural bond strength. Moreover, for all three mixing water amounts, it was found that the maximum values of bonding strength were reached after 9 days of curing, which decreased over time. The largest decreases (30–40%) were recorded after 14 days. After 21 days, these values continued to decrease, but more slowly. The final value of the ratio of bond strength to flexural strength of the mortar was similar for all amounts of mixing water and for the 28-day curing time, it oscillated around 0.2.

## 1. Introduction

Clinker products, especially solid bricks and various types of hollow units with glazed surfaces, are mainly used for the outer layers of enclosure walls in buildings [1]. These can be the outer layers of cavity or veneer walls, as well as self-supporting façade walls, which are connected to load-bearing enclosure wall structures only with appropriate anchors or frame structures, such as those made of stainless steel [2]. Due to the nature of their work in the structure and exposure to environmental influences, adequate durability of such walls and structures is required. In case of masonry façades, the permanent bond between these units and mortar must also ensure durability, in addition to the appropriate physical and mechanical parameters of the clinker elements themselves. The decisive parameter is bond strength, which determines both the strength parameters of the masonry structure, such as out-of-plane flexural strength of the masonry and in-plane shear strength of the wall, as well as any possibility of crack appearance, scratches, and spalling that may occur at the interface between the element and mortar. Thus, two types of this strength can be distinguished:

(i) flexural bond strength, which characterizes the out-of-plane flexural strength of the wall, and
(ii) shear bond strength, which determines the adhesion of mortar to the in-plane masonry elements of the wall, which is the shear strength in the direction parallel to the bed joints.

In the cases of exterior curtain walls and masonry lintels [3], the first of these strengths, namely flexural bond strength, is more important. This is related to the predominant external load on such a wall, namely wind pressure or suction, which produces a flexural effect on the wall in a plane perpendicular to its surface. However, it should also be borne in mind that studies have also shown that an increase in bond strength, while keeping mortar strength constant, also leads to an increase in the compressive strength of the wall [4].

Analyzing the literature on the subject, one can find different approaches to the determination of bond strength over the past few decades [5,6,7,8,9]. In the case of shear bond strength (e.g., [10,11,12]), it is equated with shear strength in the plane of the masonry, determined by a three-point shear test, such as in accordance with EN 1052-3:2005 [13]. This case is somewhat different with regard to flexural bond strength. For many years, a suitable way to determine this strength has been sought using various approaches, even in the form of axial tensile tests, e.g., [5,8,14]. Most of these methods involved an axial tensile test of a sample composed of two masonry units held together by a single layer of mortar. The tests mainly differed in the way the load was applied to the masonry units (through steel plates or rods glued to the bed faces or to the head faces of the units). The necessity of perfectly axial fastening of steel elements to the masonry units caused great difficulties in execution and generated additional costs. The second group consisted of tests in the four-point bending test, which required the use of test elements consisting of a minimum of eight masonry units. The results were sometimes debatable because in the case of failure in the joint outside the system of loading forces (out of the zone of constant bending moment), the value of shear forces also had to be taken into account. Methods of loading masonry panels in a plane perpendicular to the plane of the wall [15], e.g., according to the American ASTM E518/E518M—10 standard [16], are used to determine the flexural strength of masonry. There were also propositions of torsion in the plane of two masonry units connected to each other by mortar as proposed Khalaf [17]. In addition, nowadays, the axial tensile test is sometimes used to determine bond strength or, actually, adhesion between a masonry element and mortar [18]. On the other hand, the flexural strength of masonry in a plane perpendicular to its surface is determined on medium-sized panels in a four-point bending test, for example, according to EN 1052-2:2005 [19]. However, the value obtained then does not characterize the flexural bond strength understood as the adhesion of mortar to the masonry element during its detachment during out-of-plane bending, especially when considering the way the masonry elements are bonded (vertical crack failure) [20]. For years, it has been accepted that this phenomenon is best characterized by the strength/parameter referred to in the “wrench test” method. This method has been refined, calibrated, and analyzed for many years ([7,21,22,23,24]), and it is used practically all over the world for both the determination of the bond strength (adhesion) of ordinary mortars [5,23,25,26], as well as new modified mortars modified (e.g., with polymers [27]), or spray repair mortars [28]. As a result, corresponding standard approaches have been developed, which eventually took the form of the European standard EN 1052-5:2005 [29] and the American ASTM C1072-13 [30], which are now widely used virtually worldwide, often as national versions of one or the other standard.

It is obvious that many different factors, such as the rate of water absorption of brick, water suction of brick, loss of moisture in mortar (water retention value), permeability, thickness of bed joints, brick and mortar combination, tensile strength of masonry [31], and substrate surface characteristics (such as roughness, porosity, and chemical adhesion between the two materials), affect the obtained values of bond strength, which are also determined by the wrench test method [32,33]. For a given masonry unit/mortar combination, the most important factors can be considered to be those related to the migration of water from fresh mortar to the masonry element and, at a later time of hardening, from the masonry element to the mortar [34,35]. It is assumed that the loss of water from fresh mortar is related to the development of the bond strength of the mortar to the brick in the masonry. In order to explain the development of bond strength, it is necessary to particularly consider the effect of water flow on the composition and hydration conditions of the mortar–brick interface. However, many cases of unexpected bond behavior are still recorded, and current understanding of this complex phenomenon is still insufficient [32,36,37,38,39,40]. Of course, the migration of water from mortar to masonry elements and vice versa is influenced by various parameters, such as quantity of mortar water [35,36,41,42] unit absorption characteristics, initial rate of absorption (IRA) [43,44,45,46], sorptivity ([43,47,48], suction rate [49], flow and retention [38], curing time and conditions ([13,29,36,37,38,39,45,46]), and workmanship [16,31]. Other factors related to masonry elements are also important, such as the brick–mortar interface [4,43], mortar properties [7,50], mortar type [51], and composition [52], as well as the differences between parameters determined under laboratory and on-site conditions [44].

These issues, as mentioned in the introduction, are particularly relevant to the construction of exterior curtain wall (façade) layers of clinker masonry elements both in the form of on-site openwork wall masonry and assembled in the form of prefabricated façade wall panels. Considering the low absorption and absorption properties of surface-glazed clinker elements, within the framework of the presented research, the influence on the flexural bond wrench determined by the wrench test method was analyzed for two very important factors, namely the water content of the mortar and the curing time.

## 2. Materials

### 2.1. Clinker Units

**Figure 1:**
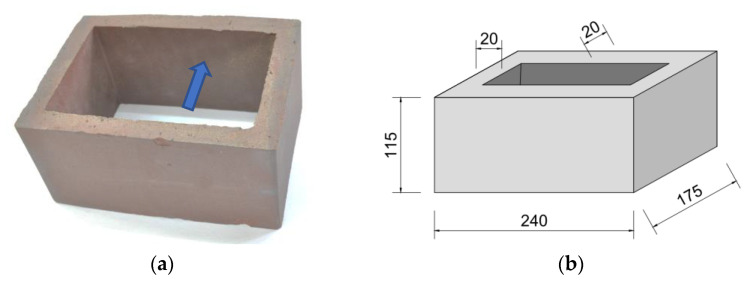
Hollow clinker unit: (**a**) view with visible slight bending of the outer rib (marked with an arrow); (**b**) nominal overall dimensions (in mm).

The façade ceramic shapes used in the present study are clinker hollow units with nominal outer dimensions of 240 × 175 × 115 mm ± 2 mm and a wall thickness of 20 mm ± 2 mm (Figure 1), designed to make the openwork outer layer of a building façade. These are not typical, commercially available façade elements. They were designed and made especially for the construction of a large academic building; therefore, the parameters specified for this type of product in EN 772-1 [53] were not fully required. Due to the shape of the units (one very large hole, covering about 65% of the element’s lateral surface) and the results of the firing process of these clinker units, the outer ribs were usually slightly bent to the outside (especially the ribs in the longitudinal direction—see Figure 1a). Their dimensional deviations, checked on 12 elements, reached even 8 mm, which caused uneven joint thicknesses during the execution of masonry test specimens because the openings of the element were in the horizontal direction and perpendicular to the plane of the wall.

The average air-dried weight of one element was 4.5 kg ± 0.02 kg. The elements were characterized by vitrification visible on the external surface and the absence of visible perforation and mechanical damage. However, it was possible to observe varying roughness, and varying levels of external surface vitrification.

The elements were ultimately intended to produce prefabricated wall panels (covering 16 or 20 hollow clinker units in one panel) with reinforcement placed in the joints. For this reason, and in view of such significant dimensional deviations confirmed, it was decided to adopt a 30 mm mortar joint thickness.

**Figure 2:**
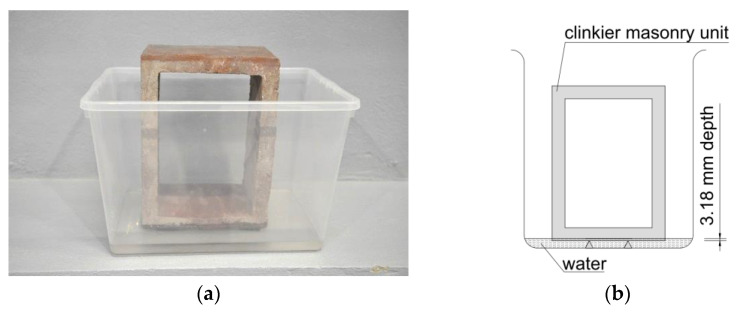
Determination of IRA: (**a**) view of the specimen during test; (**b**) scheme of the test.

In order to determine the absorbency of these units, the initial rate of absorption (IRA) was determined in accordance with ASTM C 67-17 [54]. The same storage conditions were adopted for the hollow clinker units as the materials in the construction factory, where the prefabricated wall panels were made from the same hollow clinker units. Until the test, the clinker units were stored in a room at a temperature of 24 °C ± 8 °C and a relative humidity of 30 to 70%. They were stored under these conditions until two consecutive weighings at 2 h intervals showed a weight change of no more than 0.2% in the last determined clinker units’ weight. The clinker units were placed in a plastic tray on steel supports as is shown in Figure 2b.

The water level was adjusted to 3.18 ± 0.25 mm (1/8 inch) above the contact plane of the test piece with the water. It was kept constant during the whole test (60 s). After removing the element from the water, it was carefully wiped off and weighed again. In total, five samples were tested in accordance with the recommendations of the standard [54].

The determined average value of IRA was 6.54 g/min./30 in.^2^, with a coefficient of variation CoV = 12.3%. As for the masonry clinker units, the value of IRA turned out to be slightly inflated. For these types of units, which fall into the group of low-absorption or low-suction units, the maximum IRA value should not exceed 5 g/min./30 in.^2^ [55]. The IRA value determined for the analyzed clinker units was close to the lower limit of the range, characterizing the masonry units as middle-absorption or middle-suction units. The values obtained indicate that the masonry units can absorb moisture from fresh mortar at a rapid rate and may impair bond strength [56].

### 2.2. Mortar

In this study, ready-mixed mortar (factory-ready dry mix) was used as recommended by the manufacturer for the construction of clinker masonry. According to the manufacturer’s declaration, mortar has a strength class of M10, meeting the requirements given in EN 998-2:2016-12 [57]. Due to the secrecy of the manufacturer of the mortar, its exact composition is not known. From the available information on the composition of the mortar, it appears that the main component of the binder is Portland cement, but it is not known whether it is clinker cement (without specifying which group) or Portland cement with the addition of fly ash or slag. In addition, mortar also contains calcium hydroxide (its content does not exceed 5% of the dry weight of the mixture), as well as an air-liquefying additive, a plasticizer, and sealing additives (it is not known exactly which ones were used). The sand content is at least 75% of the dry weight of the total mixture. In addition, the mortar contains an additive of Rhine trass.

The adoption of a ready-made dry mixture for the construction of masonry wall panels requires only the addition of the appropriate amount of mixing water and should ensure good quality and repeatability of the prepared mixture, as well as the façade panels made with it. In the case of the mortar used, the manufacturer recommends the use of 3.75–4.25 L per 25 kg (1 bag) of dry mix, which, after conversion, gives the recommended amount of water to be used in the range of 15% to 17% of the weight of the dry mix. Tap water was recommended and used. Given the fact that the IRA value determined for clinker masonry units slightly exceeded the upper limit for low-absorbing or low-suction units, it was decided to also analyze the use of slightly higher amounts of mixing water, in addition to the amount recommended by the mortar manufacturer. Among other things, the amount of mixing water was increased to 4.5 and 5.0 L per 25 kg of dry mix. This made it possible to achieve the good workability of the mortar needed for precast panels.

Tests on the mechanical properties of mortar were carried out in accordance with EN 1015-11:1999 [58]. The authors presented the exact results of these tests in [59]. The analyzed factors affecting the mechanical parameters of mortar were as follows:

- amount of mixing water—4 L, 4.5 L, and 5 L were adopted;
- curing time—five periods were assumed, namely 5 days, 9 days, 14 days, 21 days, and 28 days.

In [59], the values of flexural strength and compressive strength were determined and analyzed for each of the above-mentioned amounts of mixing water and each curing time. Below, Table 1 summarizes the obtained average values of these two strengths. The coefficient of variation for flexural strength was not determined because only three specimens were tested each time. Recalling these mechanical parameters of mortar from the earlier stage of research yields a comparison of the tested bond strength with the flexural strength of mortar itself.

## 3. Program and Technique

Flexural bond strength tests using the wrench test method were conducted in accordance with the European standard EN 1052-5:2005 [29]. The variable parameters in these tests were as follows:

- amount of mixing water—4 L, 4.5 L, and 5 L were adopted;
- curing time—four periods were adopted, namely 9 days, 14 days, 21 days, and 28 days.

According to the information obtained from the contractor of the prefabricated wall panels, five curing times were initially planned, namely 5, 9, 14, 21, and 28 days. The first two periods resulted from technology and the possibility to produce prefabricated wall elements; therefore, tests after 7 days were not planned. Finally, based on the results of testing mortar itself, testing the elements 5 days after their execution was abandoned due to the expected low strength of the mortar and the possibility of damaging the test element during its preparation for the wrench test, mainly while placing it in the test stand and attaching the loading arm bracket. Several attempts were made to test the models this way 5 days after they were made, but these were unsuccessful. Therefore, four curing times were finally adopted. The specimens were tested after 9, 14, 21, and 28 days of curing. The exact program is presented in the Table 2. A general method of marking of the test specimens as WT_xx_yL was adopted, where

WT stands for wrench test;

xx means curing time (in days);

yL is the amount of water used to prepare the mortar mix (in liters per 25 kg bag).

**Figure 3:**
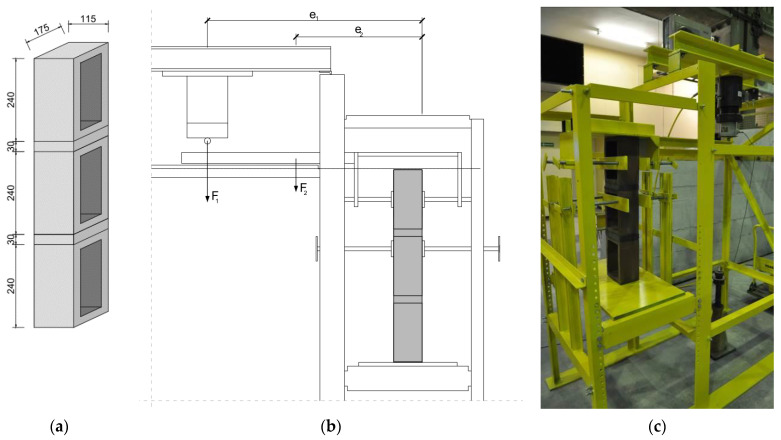
Experimental setup for bond wrench test: (**a**) shape and overall dimensions of the test specimens; (**b**) static scheme; (**c**) view of the specimen prepared for testing.

The test specimens took the form of prisms made of three clinker masonry units, in which the supporting (bed) surfaces were joined by mortar (Figure 3a). The tests’ specimens were prepared in a vertical position. Clinker units were set vertically and joined with head planes (smaller ones) due to the fact that, as previously mentioned in Section 2.1, during their manufacture (firing process), the longer sides were bent more out of plane (greater dimensional inaccuracies). Joining the elements with head planes (flatter without greater deviations when it comes to surface splicing) made it possible to achieve more or less equal joint thickness. Care of the maturing test specimens consisted of preventing excessive drying of the mortar by covering them with polyethylene film until the test. The specimens matured in this way were stored in the laboratory hall at a temperature of about +200 °C ± 20 °C and a relative humidity of about 35–60% (this was in the summer period).

Each test specimen was fixed in a test stand (the scheme of the stand is shown in Figure 3b,c) so that the force normal to the supporting surface and the bending moment were applied to the upper masonry unit. It was necessary to protect the second-top masonry unit from rotation. The lever load increased smoothly at a speed of 0.1 N/s. The force was implemented using a small hydraulic actuator. The test continued until the upper masonry unit was detached from the rest of the specimen.

Flexural bond strength is considered the value of the maximum tensile stress occurring at failure in the support plane of the upper masonry unit. According to [29], the value of individual bond strength is calculated for each individual test to the nearest 0.01 MPa from Formula [1], considering the effect on maximum tensile stresses normal to the plane of the bond, the bending moment and influence of the compression caused by the dead weight of the lever, and the clamp and the weight of the detached masonry unit:

(1) $$f_{wi}=\frac{F_{1}e_{1}+F_{2}e_{2}-2/3d{\left(F_{1}+F_{2}+\frac{W}{4}\right)}_{}}{Z},$$

where

*Z* is the section modulus of the projected plan area of the failure (in mm^3^):

(2) $$Z=\frac{bd^{2}}{6},$$

*b* is the mean width of the bed joint tested (in mm);

*d* is the mean depth of the specimen (in mm);

*e*_1_ is the distance from the applied load to the tension face of the specimen (875 mm);

*e*_2_ is the distance from the center of gravity of the lower and upper clamp from the tension face of the specimen (57.5 mm);

*F*_1_ is the applied load (read during the test, w N);

*F*_2_ is the weight of the bond wrench (262 N).

## 4. Modes of Failure

Despite the very careful arrangement of the masonry specimens at the test stand, damage to the joints (cracks or scratches in the sample) was noticed in a total of seven specimens. In all cases, they were damaged during assembly at the test stand, prior to testing.

**Figure 4:**
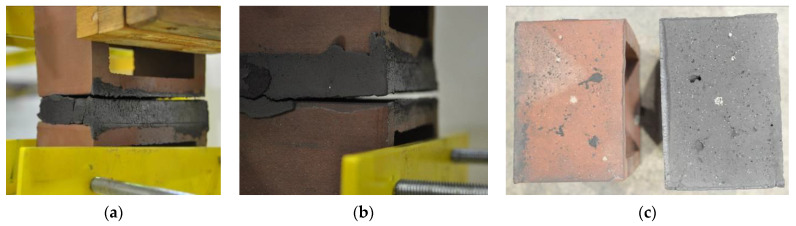
Two typical modes of failure: (**a**) cracking under the top unit; (**b**) cracking above the bottom unit; (**c**) visible lack of mortar adhesion to the surface of one clinker unit.

Very similar failure mechanisms were observed during the tests, regardless of the amount of mortar mix water used and the amount of time since the test specimens were prepared. The vast majority of the specimens (63 out of a total of 72) failed in the plane of the support (bed) joint at the interface between the mortar and the masonry unit (Figure 4a,b). These are type A1 or A2 failures, respectively, according to the classification given in [20]. It was observed that in these cases, all the mortar of the joint remained glued to one masonry unit, while the other masonry unit had only a few dirt and mortar residues as shown in Figure 4c. Information about the location of delamination (at the lower or upper masonry unit) was very important and carefully noted as the weight of the detached masonry unit with any mortar remaining on its surface is necessary to take into account when calculating flexural bond strength *f_wi_*.

In the case of the nine test specimens, it was observed that a small amount of mortar also remained on the second clinker unit. Thus, this was an intermediate mechanism between A3 (about half of the mortar of the joint adheres to the upper masonry unit and the other half to the lower) and A4 (in-plane tearing of the mortar of the joint; mortar of about ½ the thickness of the joint adheres to both planes of the masonry unit), according to the classification adopted in Annex A of the standard [29]. Finally, this failure mode was classified as delamination of the mortar in the joint, that is, type A4 failure mode, according to [29]. However, it is worth mentioning that delamination in the bed joint occurred very close to the contact with one of the masonry elements (and not around the middle of the joint thickness), so the qualification of the type of destruction was somewhat debatable.

Table 3 shows the typical modes of failure in test specimens that are most common in a given series. Of course, some specimens in a given series have a slightly different mode of failure than shown in the example. Careful visual inspection of the surfaces of masonry units after the test showed that failure due to delamination in the bed joint (assumed as type A4 modes of failure) occurred in the case of test specimens made of clinker units characterized by slightly higher surface roughness compared to the other units. This phenomenon was observed in all series regardless of the amount of mixing water used and regardless of the age of the test specimens at the time of testing. This means that any inaccuracy and/or unevenness in the glazing of the surface of the clinker units positively affected the flexural bond strength values.

In summary, therefore, it can be concluded that the amount of water used for mortar preparation and the age of the masonry specimen did not have any influence on the localization and mode of failure. The modes of failure were not associated with the type of tested series (the age of masonry specimens or the mortar water content), and were only associated with the quality of the clinker unit’s external (contact) surface (its porosity, roughness, or harshness).

## 5. Test Results and Discussion

Tests on flexural bond strength of masonry specimens made of clinker masonry units and the recommended mortar, carried out according to the standardized “wrench test” method, clearly showed that both the age of the specimens at the time of testing and the amount of water used to prepare the mortar have an effect on the results obtained. The detailed results obtained in the presented tests for each series of test pieces in the form of individual bond strength *f_wi_*, average bond strength *f_w,av_*, standard deviation *s_d_*, and coefficient of variation (*CoV*) are presented collectively in Table 4, Table 5 and Table 6, respectively.

**Figure 5:**
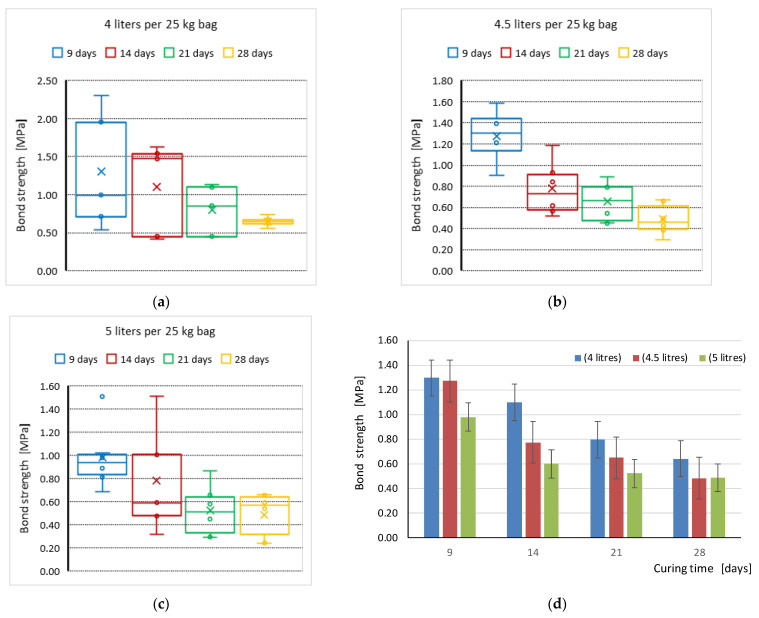
Flexural bond strength in terms of dependence with the curing time (days) of specimens: (**a**) WT_xx_4L series; (**b**) WT_xx_4.5L series; (**c**) WT_xx_5L series; and (**d**) comparison of mean values with standard errors for all tested series.

Analyzing the results in these tables, it is easy to see that the highest values of bond strength were obtained during the testing of specimens 9 days after they were made. As the curing time increased, the values of bond strength decreased and the smallest values were obtained for the period of 28 days after the test specimens were made, that is, when the mortar should have already reached its target mechanical parameters. A graphical interpretation of the changes in flexural bond strength in terms of dependence with the curing time (days) average values for each tested series is shown in Figure 5a–c, respectively. These figures show the mean values, internal and outlier points, and the calculation of the quantile, taking into account the median. Meanwhile, Figure 5d shows the comparison of the mean values with the standard errors for all tested series.

The obtained results show that as the curing time increased, the value of the bond strength decreased, and it is usually expected that it should increase with the passage of time. This is confirmed by the statement made by Groot and Labbri [38] in 1999 that “*(...) many cases of unexpected bond behavior are still registered, and apparently insight into this complex phenomenon is still incomplete.*”

The present study shows the effect of both factors on the achieved bond strength values. Increasing the amount of mixing water by about 6% and 17.5% from the maximum value recommended by the manufacturer, i.e., 3.75–4.25 L per 25 kg of dry mix, had a negative effect on the flexural tensile strength values obtained for all curing times. The curing time also had a large effect, with the highest values obtained after 9 days and the lowest obtained after the nominal curing time and mortar duration of 28 days. After analyzing the results shown in Figure 5, it is easy to see that the bond strength of the specimens tested after 28 days of curing was about two times lower. In the case of the 4.5 L series, the bond strength was even almost three times lower than the average value obtained for the samples tested after 9 days of curing. Moreover, the greatest decrease in strength occurred 14 days after the test specimens were made. After that, the situation stabilizes, and further decreases after 21 up to 28 days become smaller and smaller.

In order to determine whether a given factor, i.e., the amount of mixing water and curing time, has a statistically significant effect on the analyzed feature (the value of the bond strength), appropriate ANOVA (assuming a significance level of 0.05) was performed. The analysis of the effect of curing time on the bond strength values showed the following for individual series (with mixing water content of 4, 4.5, and 5 L) that the curing time:

(i) for the WT_xx_4L series—had no significant effect (F = 1.575 < F_cr_ = 3.239);
(ii) for the WT_xx_4.5L series—had a significant impact (F = 10.613 > F_cr_ = 3.160);
(iii) for the WT_xx_5L series—had a significant impact (F = 3.452 > F_cr_ = 3.127).

This means that only for the series in which 4 L of mixing water were used was there no significant impact of curing time on the analyzed bond strength (there is no observed significant effect between groups representing individual curing times).

The effect of the factor related to the amount of mixing water on the bond strength values for individual curing times was also analyzed using ANOVA, assuming a significance level of 0.05. In this case, convergent results were obtained. In all four cases (for 9, 14, 21, and 28 days), there was no reason to reject the null hypothesis that there was no significant effect of this factor on the analyzed feature (in all cases, the F-statistic value was less than F_cr_).

Of course, as is well known, a great many factors affect the values of bond strength [33,52,59], including mortar physic-mechanical parameters [7,53,56,60,61]. In the processes presented and analyzed below, the value of IRA is, of course, very important, but so is the connection with the porosity of masonry elements (number of open pores and total number of pores) and the size and number of capillaries [62], also in relation to mortar, which is equally important [50].

The slightly elevated IRA value found in the study, relative to the maximum for clinker units, resulted in slightly different behavior of mortar in the contact layer with the surface of these masonry units. It is generally believed that the loss of water from fresh mortar is related to the development of the bond force between the mortar and the masonry unit in the masonry. When explaining the development of the bonding force, the effect of water flow on the composition and hydration conditions of the mortar–brick interface should particularly be considered. In the presented tests, in the case of the amount of mixing water recommended by the mortar manufacturer (4 l per 25 kg bag) when using hollow clinker masonry units and due to the slightly higher absorbency of these units, a slightly larger amount of water was drained out of the fresh mortar in the initial hardening phase. As a result, the amount of water was at a sufficient level for proper hydration and setting process in mortar. However, the increased absorption of water in the subsequent periods caused further drainage of water from the mortar, which somewhat disrupted the hardening process. As a result, bond strength decreased over time. It should be borne in mind that not only the mere flow of water from the mortar to the brick, which takes place immediately after the mortar comes into contact with the brick (capillary rise in the masonry unit), but also the reverse flow of water from the element to the mortar, which takes place after the mortar has been compacted and pre-hydrated, can significantly affect adhesion between the masonry unit and the mortar surface and, consequently, bond strength.

On the other hand, the increased, as opposed to the recommended, content of mixing water in the preparation of mortar and the use of it to join clinker units with a glazed surface must lead to disturbances in the conditions of setting and hardening of mortar in the mortar joints. However, the clinker unit, despite its slightly elevated IRA value, is not very absorbent (absorbency of less than 6%), so it is unable to absorb excess water from fresh mortar. As a result, a proper crystalline network cannot be formed at the time of setting as water molecules that were not chemically bound in the pozzolanic process remain. Initially, excess water may be associated with slightly increased adhesion of the mortar to the surface of the masonry elements, but as the hardening process develops and the mortar dries, the unbound water molecules are partly removed and partly built into the structure of the mortar’s crystalline network. Increased shrinkage associated with mortar drying is then observed. The result is a deterioration in the mechanical properties of the hardened mortar, especially its adhesion to the surface of the ceramic masonry unit. In addition, it can also result in lower bond strength values. This effect is particularly evident during the drying process of mortar; the more mature the sample is, the greater the negative effect of drying shrinkage.

**Figure 6:**
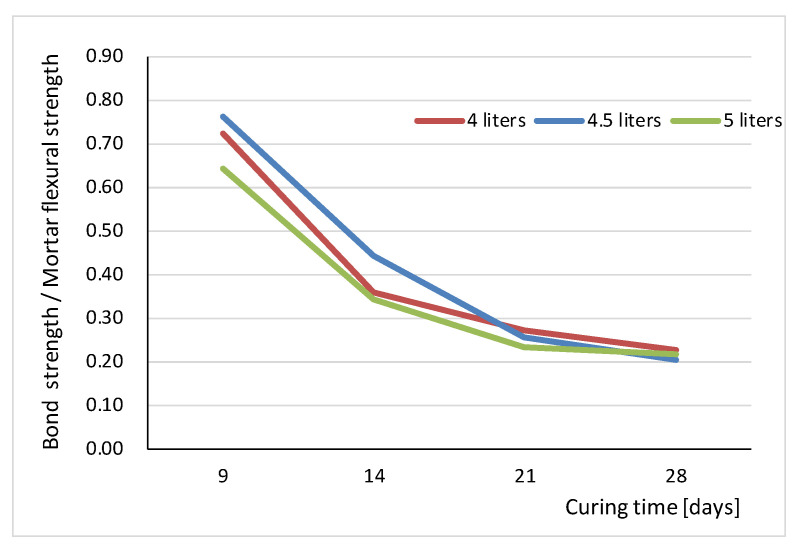
Comparison of average values of bond strength with flexural strength of the mortar for all tested series.

In addition, analysis of the properties of mortar itself (compressive strength and flexural strength) presented in Table 2 [59] shows that the 28-day parameters are significantly lower than the parameters after 14 and 21 days of curing time. This phenomenon can be explained, at least in part, from the point of view of the interaction of masonry units and mortar. In this context, it is interesting to compare the obtained values of flexural bond strength with the flexural strength of the mortar itself, as is graphically shown in Figure 6.

It can be seen that the ratio of the average value of bond strength to the flexural strength of mortar is very similar in all the series studied for all three amounts of mixing water and the curing times analyzed. The highest value of the order of 0.7 on average occurred at 9 days, and then quickly decreased to reach the lowest value after 28 days, which was just over 0.22 and almost identical for all three series. Therefore, it can be assumed that in the presented tests, after 28 days, the value of flexural strength of mortar is about five times higher than bond strength. This leads to the conclusion that mainly IRA and the associated porosity of masonry elements influenced the reduction in bond strength.

The research presented above shows that the issue of the influence on the obtained values of flexural bond strength determined using the wrench test method of the two main factors analyzed, namely the amount of mixing water used to prepare the mortar mixture and the curing time, is still not fully understood. It still requires further research and analysis, especially with regard to the microstructure of both the mortar itself and the masonry elements, as well as the physic-chemical reactions taking place in the setting and hardening mortar in terms of additives, which are usually a component of ready mixes and are not specified by the mortar manufacturer.

## 6. Summary and Conclusions

This article presents the results of experimental studies on the effect of the amount of mortar water used in the preparation of mortar and the curing time on flexural bond strength determined using the wrench test method. The tests concerned clinker units with unusual perforation (one large horizontal hole), intended for façade curtain walls. Furthermore, mortar in the form of a factory-ready mix was used, which is recommended for bricklaying clinker units.

Considering all the experimental results obtained and the analyses presented above, the following conclusions can be made:

- The presented experimental studies showed a significant, negative impact of both the increase in the amount of mixing water in relation to the recommended value and the curing time on the values of bonding strength obtained when using hollow clinker units and the mortar recommended for them.
- In the analyzed case, the maximum values of bonding strength were found after 9 days of curing, which decreased over time. The largest decreases (30–40%) were recorded after 14 days. After 21 days, these values continued to decrease, but they did so more slowly.
- The tested elements after 28 days were characterized by a 50% decrease in bond strength for the recommended average amount of water, i.e., 4 L per 25 kg of the mix and 18% more mixing water, as well as an almost 60% decrease when the amount of water increased by 6%.
- The ratio of bond strength to flexural strength of the mortar was similar for all amounts of mixing water for each curing time. It was the highest for the values obtained after 9 days (of the order of 0.7) and decreased in subsequent periods. The final value after 28 days was also very similar and oscillated around 0.2.
- The conducted ANOVA showed that there was no statistically significant effect of the curing time on the tested bond strength, only for the series in which 4 L of mixing water was used. However, in the case of the influence of the amount of mixing water on the values of bonding strength for individual hardening times, there were no grounds to reject the null hypothesis that this factor had no significant effect on the analyzed feature.

Summarizing up the above conclusions, it can be stated, as already mentioned, that one must be quite careful when using clinker units intended for façade walls, even when using mortar elements recommended for this type. It may turn out that the bond strength a few days after completion of the wall is much higher than in subsequent periods.

In the presented studies, non-standard (specially designed and manufactured for the needs of a large investment) hollow clinker units and dedicated mortar in the form of a factory-ready mix were used. Therefore, in order to better understand the analyzed issues, it is necessary to conduct further research on various ceramic façade units (solid and vertically perforated bricks) offered on the construction market that meet the requirements of the standard [53] with the use of factory-ready-made mortar mixtures recommended for this type of masonry units.

## Figures and Tables

**Table 1:** Summary of mortar test results [59].

Mixing Water (L/25 kg bag)	Strength (MPa)	Curing Time (Days)			
		9	14	21	28
4.0	flexural	1.68	2.92	2.94	2.91
	compressive (CoV)	9.22 (10.2%)	10.01 (3.1%)	10.84 (7.1%)	10.43 (7.0%)
4.5	flexural	1.67	1.50	2.56	2.39
	compressive (CoV)	8.15 (6.5%)	9.06 (4.9%)	10.11 (6.9%)	9.20 (3.8%)
5.0	flexural	1.51	1.76	2.25	2.15
	compressive (CoV)	6.95 (6.3%)	9.08 (6.5%)	9.07 (5.4%)	8.39 (6.9%)

**Table 2:** Wrench test program.

Series	Curing Time (Days)	Number of Samples	Mixing Water Amount per 25 kg of Dry Mixture		
			4 L	4.5 L	5 L
WT-09_4L	9	6	X		
WT-14_4L	14	6	X		
WT-21_4L	21	6	X		
WT-28_4L	28	6	X		
WT-09_4.5L	9	6		X	
WT-14_4.5L	14	6		X	
WT-21_4.5L	21	6		X	
WT-28_4.5L	28	6		X	
WT-09_5L	9	6			X
WT-14_5L	14	6			X
WT-21_5L	21	6			X
WT-28_5L	28	6			X

**Table 3:** Exemplary, typical modes of failure of tested specimens in individual series. The type of failure letter designation is in accordance with the classification of modes of failure given in EN 1052-5 [29].

	Curing Time (Days)			
	9 Days	14 Days	21 Days	28 Days
Series WT_xx_4L	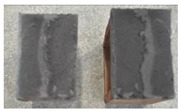	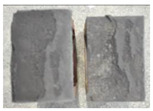	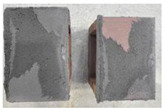	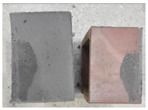
	WT-09-4_4L (A4)	WT-14-2_4L (A4)	WT-21-3_4L (A2)	WT-28-1_4L (A2)
	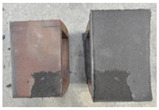	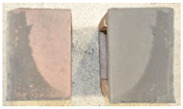	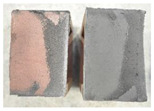	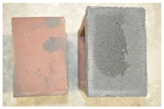
	WT-09-6_4L (A2)	WT-14-6_4L (A1)	WT-21-6_4L (A1)	WT-28-3_4L (A1)
Series WT_xx_4.5L	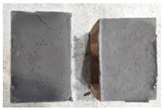	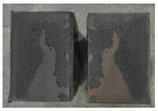	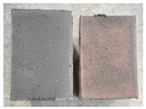	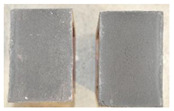
	WT-09-2_4.5L (A4)	WT-14-6_4.5L (A4)	WT-21-2_4.5L (A2)	WT-28-3_4.5L (A4)
	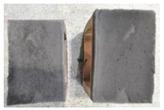	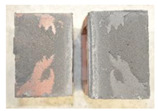	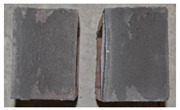	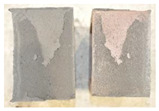
	WT-09-6_4.5L (A4)	WT-14-4_4.5L (A1)	WT-21-4_4.5L (A4)	WT-28-6_4.5L (A2)
Series WT_xx_5L	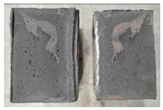	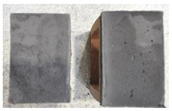	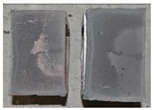	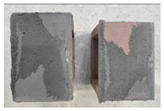
	WT-09-1_5L (A4)	WT-14-4_5L (A4)	WT-21-3_5L (A4)	WT-28-3_5L (A4)
	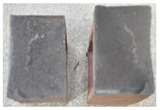	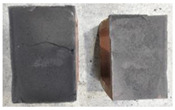	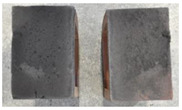	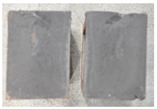
	WT-09-4_5L (A4)	WT-14-5_5L (A4)	WT-21-6_5L (A4)	WT-28-4_5L (A4)

Key to failure modes presented in this table (according to [29]): A1—Failure at interface between mortar and upper unit; A2—Failure at interface between mortar and lower unit; A4—Tension failure within mortar bed.

**Table 4:** Bond strength results for series using 4 L of mixing water.

Specimen	f_wi_ [MPa]	f_w,av_ (±s_d_) [MPa]	s_d_ [MPa]	CoV [%]
WT-09-1_4L	0.709	1.299 (±0.782)	0.782	60%
WT-09-2_4L	0.993			
WT-09-3_4L	^*)^			
WT-09-4_4L	2.300			
WT-09-5_4L	1.951			
WT-09-6_4L	0.541			
WT-14-1_4L	0.411	1.099 (±0.613)	0.613	56%
WT-14-2_4L	1.627			
WT-14-3_4L	1.472			
WT-14-4_4L	^*)^			
WT-14-5_4L	0.450			
WT-14-6_4L	1.537			
WT-21-1_4L	0.450	0.797 (±0.335)	0.335	42%
WT-21-2_4L	1.136			
WT-21-3_4L	1.097			
WT-21-4_4L	^*)^			
WT-21-5_4L	0.851			
WT-21-6_4L	0.450			
WT-28-1_4L	0.670	0.644 (±0.067)	0.067	10%
WT-28-2_4L	0.644			
WT-28-3_4L	0.735			
WT-28-4_4L	^*)^			
WT-28-5_4L	0.553			
WT-28-6_4L	0.618			

^*)^—the specimen was damaged prior to testing.

**Table 5:** Bond strength results for series using 4.5 L of mixing water.

Specimen	f_wi_ [MPa]	f_w,av_ (±s_d_) [MPa]	s_d_ [MPa]	CoV [%]
WT-09-1_4.5L	0.903	1.275	-	-
WT-09-2_4.5L	1.213			
WT-09-3_4.5L	1.588			
WT-09-4_4.5L	^*)^			
WT-09-5_4.5L	^*)^			
WT-09-6_4.5L	1.394			
WT-14-1_4.5L	0.838	0.776 (±0.258)	0.258	33%
WT-14-2_4.5L	1.187			
WT-14-3_4.5L	0.618			
WT-14-4_4.5L	0.515			
WT-14-5_4.5L	0.566			
WT-14-6_4.5L	0.929			
WT-21-1_4.5L	0.541	0.653 (±0.195)	0.195	30%
WT-21-2_4.5L	0.890			
WT-21-3_4.5L	0.450			
WT-21-4_4.5L	0.450			
WT-21-5_4.5L	0.786			
WT-21-6_4.5L	0.799			
WT-28-1_4.5L	0.424	0.487 (±0.151)	0.151	31%
WT-28-2_4.5L	0.385			
WT-28-3_4.5L	0.489			
WT-28-4_4.5L	0.657			
WT-28-5_4.5L	0.295			
WT-28-6_4.5L	0.670			

^*)^—the specimen was damaged prior to testing.

**Table 6:** Bond strength results for series using 5 L of mixing water.

Specimen	f_wi_ [MPa]	f_w,av_ (±s_d_) [MPa]	s_d_ [MPa]	CoV [%]
WT-09-1_5L	0.890	0.983 (±0.286)	0.286	29%
WT-09-2_5L	1.019			
WT-09-3_5L	0.683			
WT-09-4_5L	0.980			
WT-09-5_5L	0.812			
WT-09-6_5L	1.511			
WT-14-1_5L	0.592	0.603 (±0.254)	0.254	42%
WT-14-2_5L	^*)^			
WT-14-3_5L	0.476			
WT-14-4_5L	1.006			
WT-14-5_5L	0.321			
WT-14-6_5L	0.618			
WT-21-1_5L	0.450	0.523 (±0.222)	0.222	42%
WT-21-2_5L	0.295			
WT-21-3_5L	0.864			
WT-21-4_5L	0.295			
WT-21-5_5L	0.657			
WT-21-6_5L	0.579			
WT-28-1_5L	0.592	0.489 (±0.195)	0.195	40%
WT-28-2_5L	0.541			
WT-28-3_5L	0.657			
WT-28-4_5L	0.243			
WT-28-5_5L	0.243			
WT-28-6_5L	0.657			

^*)^—the specimen was damaged prior to testing.

## Data Availability

Data are available on request.
